# Quantification and homogenization of image noise between two CT scanner models

**DOI:** 10.1002/acm2.12798

**Published:** 2019-12-20

**Authors:** Samuel A. Einstein, Xiujiang John Rong, Corey T. Jensen, Xinming Liu

**Affiliations:** ^1^ Department of Imaging Physics The University of Texas MD Anderson Cancer Center Houston TX USA; ^2^ Department of Diagnostic Radiology The University of Texas MD Anderson Cancer Center Houston TX USA; ^3^ Present address: Department of Radiation Safety WellSpan York Hospital York PA USA

**Keywords:** computed tomography, image noise, image quality, noise power spectrum

## Abstract

Feedback from radiologists indicated that differences in image appearance and noise impeded reading of post‐contrast computed tomography (CT) scans from an updated CT scanner that was recently added to a fleet of existing scanners from the same vendor, despite using identically named reconstruction algorithms. The goals of this work were to quantify and possibly standardize image quality on the new and an existing scanner using phantom images. Three months of daily quality control images were analyzed to determine the mean CT number and noise magnitude in a water phantom. Next, subtraction images from the uniformity section of an American College of Radiology CT phantom were used to generate noise power spectra for both scanners. Then, a semi‐anthropomorphic liver phantom was imaged with both scanners in triplicate using identical body protocols to quantify differences CT number and noise magnitude. Finally, the scanner dependence of CT number and noise magnitude on material attenuation was quantified using a multi‐energy CT phantom with 15 material inserts. Significant differences between scanners were determined using a paired or Welch's *t* test as appropriate. In daily quality control images, the new scanner exhibited slightly higher CT number (0.697 vs. 0.412, *P* < 0.001, n = 85) and slightly lower noise magnitude (4.85 vs. 4.94, *P* < 0.001, n = 85). Measured NPS was not significantly different between the existing and new scanners. Interestingly, it was observed that the noise magnitude from the new scanner increased with increasing material attenuation in both the liver (*P* = 0.008) and multi‐energy (*P* < 0.001) phantoms. Using an alternate reconstruction algorithm with the new scanner eliminated this deviation at high material attenuations. While standard noise evaluation in a water phantom was unable to discern differences between the scanners, more comprehensive testing with higher attenuation materials allowed for the characterization and homogenization of image quality.

**Abbreviations**
DQC: daily quality control
HU: Hounsfield unit
NPS: noise power spectrum

## 1 INTRODUCTION

**Figure 1:**
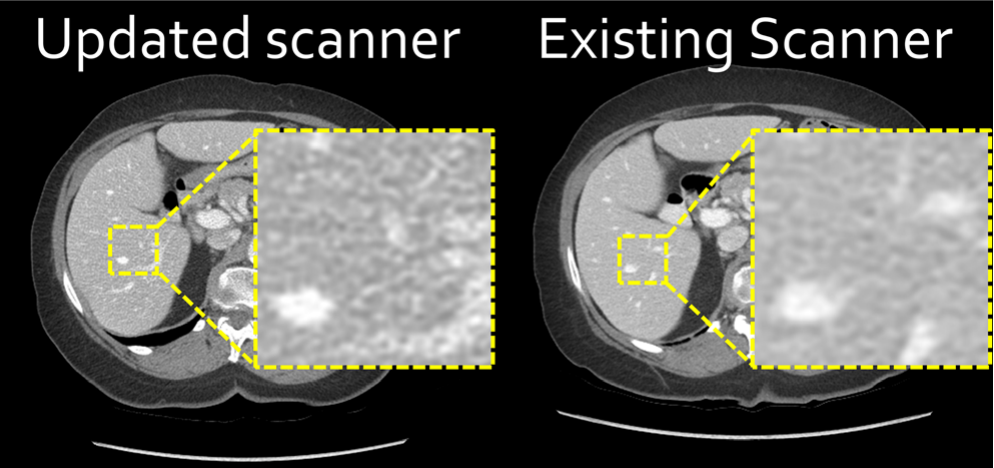
Despite using identically named standard reconstruction algorithms, the updated computed tomography (CT) scanner produced images (left) with a difference in noise appearance compared to the existing CT scanner (right), which impeded reading of body CT scans.

Computed tomography (CT) scanner technology continues to advance with developments such as multi‐detector CT; faster rotation times; radiation dose reduction through current, voltage, and organ dose modulation; and iterative reconstruction techniques. These advancements create challenges in harmonizing image quality and appearance from a large fleet of clinical scanners. Several groups, however, have quantified and homogenized image appearance on similar scanner models using uniform sections of water or water‐equivalent materials.^1^, ^2^, ^3^ At our institution, feedback from radiologists indicated that differences in image appearance and noise on a new CT scanner we recently added to our fleet impeded reading of post‐contrast scans (Fig. 1). Interestingly, image quality and noise from this scanner were quantified during acceptance testing and determined to be equivalent to existing scanners by typical image quality metrics. Additionally, these scanners were from the same vendor and expected to have similar image appearance.^1^ The goals of the present work were to quantify and standardize image quality on the new and an existing scanner using phantom images and identify new image quality metrics that should be included in acceptance testing.

## 2 METHODS

### 2.1 Daily quality control (DQC) analysis

**Figure 2:**
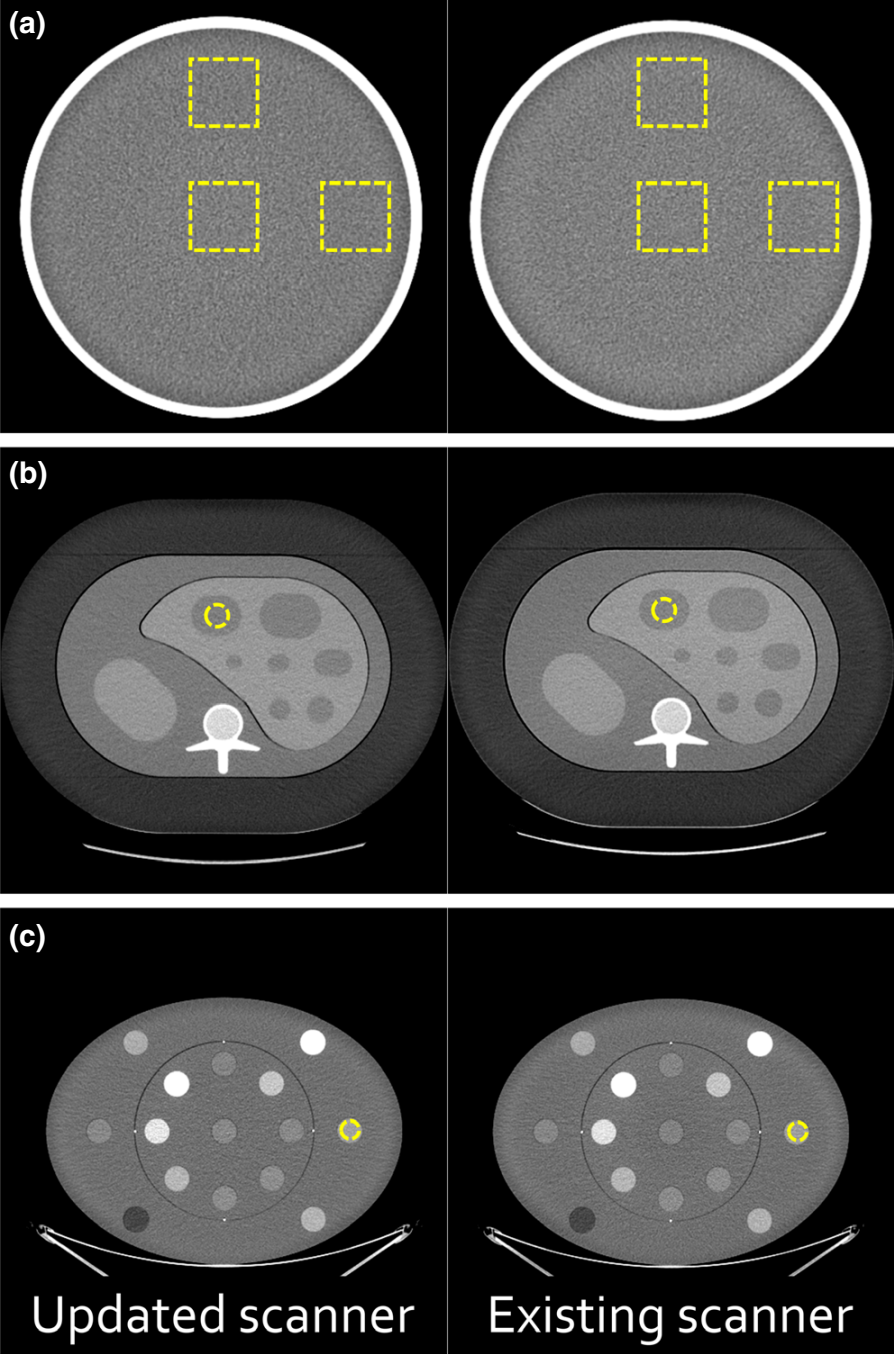
Image quality of the updated and existing scanners was measured using (a) the manufacturer‐provided daily quality control phantom, (b) a semi‐anthropomorphic liver phantom, and (c) a multi‐energy computed tomography (CT) phantom with 15 inserts. A single example region of interest is shown for each image.

Image quality from the new (Revolution HD; GE Healthcare, Waukesha, WI, USA) and an existing scanner (Revolution 750 HD GSI; GE Healthcare) were quantified using daily quality control (DQC) data acquired over 90 days using the manufacturer‐provided QC phantom. Acquisition parameters included 120 kVp, 335 mA tube current, 0.4 s rotation time, 0.516 pitch, head filter, 5 mm image thickness, Standard reconstruction, and 22 cm display field‐of‐view (DFOV). The average CT number and standard deviation of CT number (noise magnitude) were measured within three regions of interest [Fig. 2(a)]. Additionally, a single acquisition of the DQC phantom was reconstructed on both scanners using the Standard (both) and Soft (updated only) reconstruction algorithms to visually examine the influence of the reconstruction on the spatial resolution of the final images.

### 2.2 Noise power spectrum (NPS) analysis

NPS was calculated using subtraction images acquired in the uniformity section of the American College of Radiology CT phantom (Gammex Inc., Middleton, WI, USA). Images were acquired using 120 kVp, three different fixed tube currents (65, 260, and 650 mA), 0.5 s rotation time, 0.516 pitch, large body filter, 0.625 mm image thickness, and 25 cm DFOV. Images from both scanners were reconstructed with both the Standard and Soft reconstruction algorithms. Images obtained from the new scanner and Soft reconstruction algorithm were both left unmodified and additionally post‐processed with edge enhancement (E1). The NPS was generated using a previously validated script.^4^ The peak values of the NPS profiles and the corresponding spatial frequencies were calculated and compared.

### 2.3 Semi‐anthropomorphic phantom analysis

Next, image quality was evaluated in a semi‐anthropomorphic liver phantom (QRM GmbH, Möhrendorf, Germany). This phantom (30 cm × 20 cm × 10 cm, resin base) featured materials representing liver, two types of lesions (hyper‐ and hypointense), surrounding abdomen, spleen, cortical bone, and marrow. An additional extension ring simulating fat (5 cm wall thickness) was used to increase phantom realism. Representative CT images of the phantom (with an example region of interest selection) and Hounsfield unit (HU) values for the different materials are provided in Fig. 2(b) and Table S1, respectively. Identical clinical protocols for abdominal imaging were used to quantify differences in CT number and noise magnitude. Acquisition parameters included 120 kVp, tube current modulation (480 mA was the typical value for the phantom on both systems), 0.5 s rotation time, 0.516 pitch, large body filter, 2.5 mm image thickness (0.625 × 64 detector element configuration), and 40 cm DFOV. Images from the existing scanner were reconstructed with the Standard algorithm while images from the new scanner were reconstructed with both the Standard and Soft reconstruction algorithms. No image post‐processing was performed.

### 2.4 Multi‐energy CT phantom analysis

Finally, image quality was evaluated in a multi‐energy CT phantom [Fig. 2(c); Gammex Inc.]. This phantom contained 15 insert rods simulating a variety of materials and concentrations.^5^ Identical axial body protocols were used to quantify differences in CT number and noise magnitude. Acquisition parameters included 120 kVp, 400 mA, 0.8 s rotation time, large body filter, 2.5 mm image thickness, and 50 cm DFOV. Again, images from the existing scanner were reconstructed with the Standard algorithm while images from the new scanner were reconstructed with both the Standard and Soft reconstruction algorithms and without image post‐processing. To determine the effects of tube current and rotation time on image quality, another set of axial images were acquired with constant mAs, but varying mA/rotation time (320/1.0, 400/0.8, 460/0.7, 535/0.6, 640/0.5, and 750/0.4 s). Additionally, material‐specific plots of noise magnitude versus material HU were generated for the insert rods containing varying concentrations of iodobenzene (2, 5, and 15 mg/mL), calcium carbonate (50 and 100 mg/mL), and ferric oxide (1.03, 1.07, and 1.13 relative electron density). The slopes of the fitted straight lines were then compared to determine the effect of material on equipment performance.

### 2.5 Statistics

Significant differences in measured values were determined using either paired or Welch's *t* test, as appropriate. Differences in fitted line slopes were analyzed with extra‐sum‐of‐squares F tests (Prism 7.03; GraphPad Software, San Diego, CA, USA). Differences were considered significant if *P* < 0.05.

## 3 RESULTS

### 3.1 Daily quality control analysis

In DQC images, the new scanner exhibited slightly higher CT number (0.697 vs. 0.412, *P* < 0.001, n = 85) and slightly lower noise magnitude (4.85 vs. 4.94, *P* < 0.001, n = 85). Spatial resolution was similar between the scanners and reconstruction algorithms, though slightly improved with the Standard reconstruction (Fig. S1).

### 3.2 Noise power spectrum analysis

Measured NPS was not significantly different between the existing and new scanners. The Soft reconstruction and soft reconstruction plus edge enhancing post‐processing (E1) decreased and increased peak noise, respectively, as expected. The spatial frequency corresponding to the peak of the NPS profile was slightly decreased by the soft reconstruction without post‐processing, but not modified by scanner model or mA. Quantitative NPS results are available in Supporting Information.

### 3.3 Semi‐anthropomorphic phantom analysis

**Figure 3:**
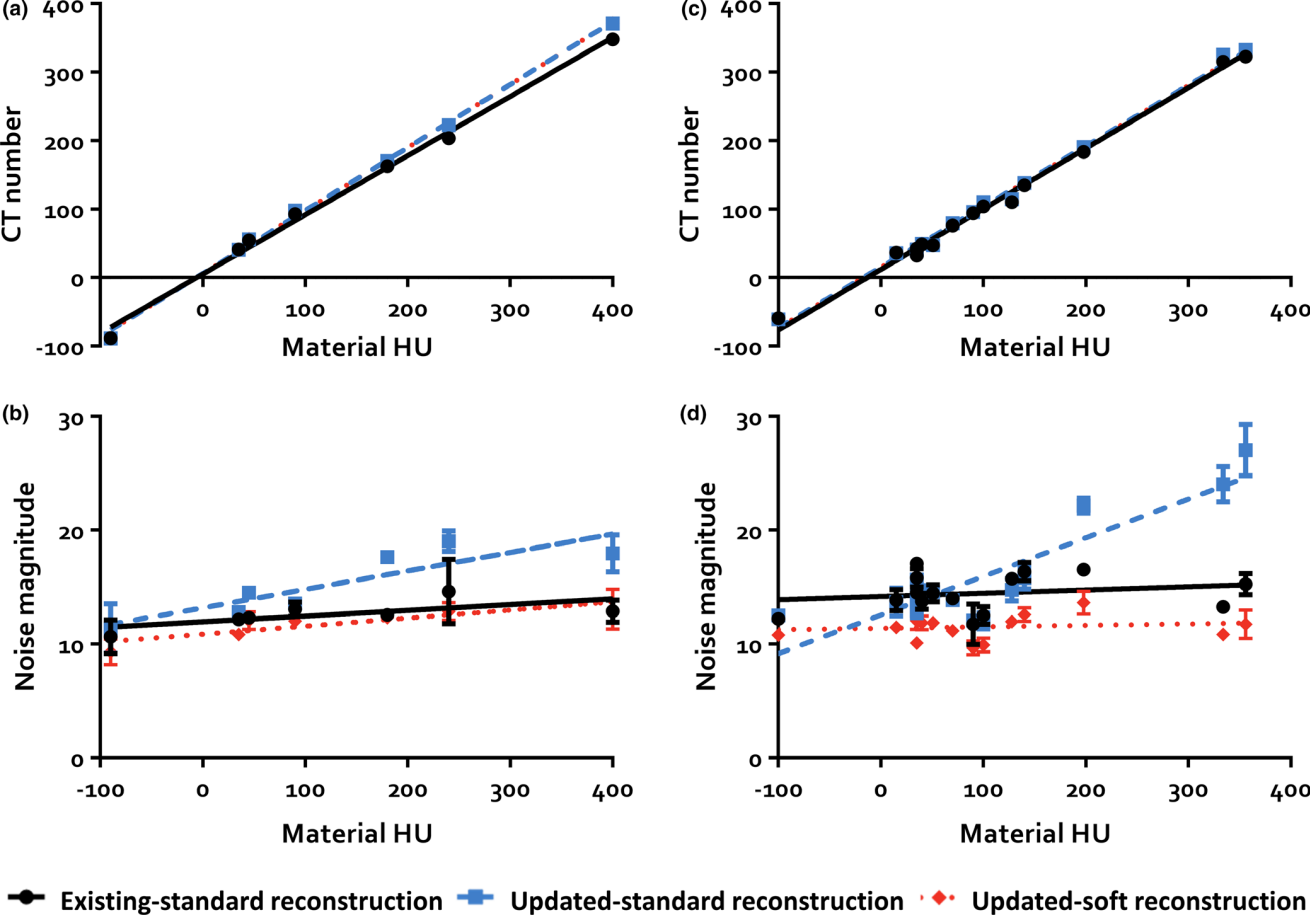
Results of the quantitative image quality analysis of the semi‐anthropomorphic liver and multi‐energy computed tomography (CT) phantoms. (a) In the liver phantom, CT numbers were similar between all scanners and reconstruction algorithms. (b) The new scanner exhibited overall higher noise magnitude than the existing scanner when utilizing the standard reconstruction and the difference in noise magnitude was dependent on material Hounsfield units (HU). This change in noise magnitude with respect to material attenuation was obviated by utilizing the soft reconstruction algorithm on the new scanner. (c) In the multi‐energy CT phantom, the mean measured CT number was slightly higher in the new scanner and (d) the difference in noise magnitude was again dependent on material HU. Utilizing the soft reconstruction algorithm on the new scanner again eliminated this deviation. This change in CT number and noise magnitude with HU was found to be independent of tube current/rotation time and material. Error bars show the standard deviations of the measurements for data points with standard deviations large enough to be visualized.

In the liver phantom images, CT numbers were similar between both scanners and both reconstruction algorithms [*P* = 0.181; Fig. 3(a); Table S1]. The new scanner exhibited higher noise magnitude (averaged over all materials) than the existing scanner when both utilized Standard reconstructions (15.3 vs. 12.6, *P* = 0.007). Interestingly, the difference in noise magnitude was dependent on material HU [Fig. 3(b); Table S2]. The change in noise magnitude with respect to material attenuation was significantly higher in the new scanner compared to the existing system (*P* = 0.008). Utilizing the Soft reconstruction algorithm on the new scanner, however, eliminated this deviation [*P* = 0.496; Fig. 3(b); Table S2].

### 3.4 Multi‐energy CT phantom analysis

The mean measured CT number averaged over all materials was slightly higher in the new scanner [105 vs. 102, *P* < 0.001; Fig. 3(c); Table S3] and the noise magnitude was increased as well (16.0 vs. 14.5, *P* = 0.022). Again, the difference in noise magnitude was dependent on material HU [Fig. 3(d); Table S4]. The change in noise magnitude with respect to material attenuation was significantly higher in the new scanner compared to the existing system (*P* < 0.001). Utilizing the Soft reconstruction algorithm on the new scanner again eliminated this deviation [*P* = 0.566; Fig. 3(d); Table S4]. This change in CT number or noise magnitude with HU was found to be independent of tube current/rotation time (CT number: *P* = 0.999, noise magnitude: *P *= 0.970) and material (CT number: *P* = 0.153, noise magnitude: *P* = 0.248), though the analyses of the material data lacked statistical power.

## 4 DISCUSSION

Increased image noise impeded clinical interpretation of post‐contrast CT scans from an updated CT scanner that was recently added to a fleet of existing scanners from the same vendor. The goals of this work were to quantify and standardize image quality on the new and an existing scanner using phantom images and determine why this issue was not discovered upon acceptance testing, which used conventional techniques employing water or water‐equivalent uniform sections of phantom to quantify image noise.^1^, ^2^, ^3^, ^4^

In this study, conventional image noise measurements within the uniform sections of the manufacturer‐provided and American College of Radiology phantoms demonstrated either equivalence between the two systems or differences too small to explain the observed phenomenon. During more advanced testing with a semi‐anthropomorphic liver phantom, it was observed that the image regions corresponding to higher HU materials exhibited higher noise appearance. This effect was again observed and more precisely quantified using a multi‐energy CT phantom. This phenomenon was influenced by neither fast rotation times nor reduced tube current (which could result from the use of tube‐current modulation). Using the Soft reconstruction algorithm on the new scanner prevented the increase in image noise with increasing material HU and reduced noise magnitude to a value below the existing scanner using the Standard reconstruction while maintaining adequate spatial resolution.

This study was limited by including only a single new scanner and existing scanner as well as only testing two approaches to reconstruction. Nevertheless, efforts to match image quality were successful with positive clinical feedback. This HU dependence of noise magnitude explained why radiologist feedback was primarily limited to post‐contrast images and illustrated the importance of using more than a simple water phantom to evaluate image noise.

## CONFLICT OF INTEREST

No conflicts of interest.
