# Supplementary material for: Quantification and homogenization of image noise between two CT scanner models
Source: J Appl Clin Med Phys. 2019 Dec 20;21(1):174–8. doi: 10.1002/acm2.12798 (PMC6964752; doi:10.1002/acm2.12798)
Supplement: Supplementary file 1 [file ACM2-21-174-s001.pdf]

Table S1: CT number measurements for objects within the semi-anthropomorphic liver phantom for the existing CT scanner (Standard reconstruction) and updated CT scanner (Standard and Soft reconstructions).

Object	HU	Existing-Standard			Updated-Standard			Updated-Soft		
**Fat**	-90	-88.4	±	0.88	-88.9	±	1.4	-88.4	±	0.5
**Abdomen**	35	41.2	±	6.55	40.5	±	1.4	39.8	±	1.0
**Nodule 1**	45	54.3	±	0.86	56.1	±	0.3	56.9	±	0.8
**Spleen**	90	92.7	±	4.32	97.6	±	5.6	97.4	±	6.2
**Nodule 2**	180	162.7	±	0.67	170.1	±	0.1	169.8	±	0.3
**Marrow**	240	203.6	±	9.55	222.6	±	6.7	222.4	±	6.7
**Cortical bone**	400	348.0	±	4.38	371.1	±	7.2	371.3	±	7.6

HU, manufacturer-provided Hounsfield unit values for the object at 120 kVp.

Table S2: CT number standard deviation (noise) measurements for objects within the semi-anthropomorphic liver phantom for the existing CT scanner (Standard reconstruction) and updated CT scanner (Standard and Soft reconstructions).

Object	HU	Existing-Standard			Updated-Standard			Updated-Soft		
**Fat**	-90	10.7	±	1.48	11.4	±	2.2	9.3	±	1.1
**Abdomen**	35	12.2	±	0.21	12.8	±	0.1	10.9	±	0.2
**Nodule 1**	45	12.3	±	0.14	14.5	±	0.1	12.1	±	0.8
**Spleen**	90	13.1	±	0.64	13.6	±	0.1	12.0	±	0.3
**Nodule 2**	180	12.6	±	0.21	17.7	±	0.4	12.3	±	0.4
**Marrow**	240	14.6	±	2.83	19.1	±	0.9	12.9	±	0.8
**Cortical bone**	400	12.9	±	0.99	18.0	±	1.6	13.1	±	1.8

HU, manufacturer-provided Hounsfield unit values for the object at 120 kVp.

Table S3: CT number measurements for objects within the multi-energy CT phantom for the existing CT scanner (Standard reconstruction) and updated CT scanner (Standard and Soft reconstructions).

Object	HU	Existing-Standard			Updated-Standard			Updated-Soft		
**Adipose**	-100	-59.4	±	0.33	-60.9	±	0.4	-60.5	±	0.3
**Brain**	15	36.3	±	0.21	35.8	±	0.3	35.6	±	0.3
**Soft tissue**	35	39.3	±	0.54	41.2	±	1.0	41.3	±	0.8
**Soft tissue**	35	42.2	±	1.82	41.1	±	0.4	41.2	±	0.3
**Soft tissue**	35	32.3	±	1.28	39.5	±	0.9	39.5	±	1.2
**Blood**	40	48.4	±	0.56	49.3	±	0.9	49.8	±	1.0
**Iodine (2 mg/mL)**	51	47.4	±	0.46	47.3	±	0.4	47.6	±	0.4
**Blood clot (normal)**	70	76.0	±	1.44	79.4	±	1.4	78.7	±	0.7
**Iodine enhancement (neuro)**	90	94.0	±	0.93	95.8	±	0.6	96.1	±	1.1
**Blood clot (extreme)**	100	104.1	±	1.09	110.2	±	0.2	108.9	±	0.6
**Iodine (5 mg/mL)**	128	110.4	±	0.64	114.4	±	0.5	113.5	±	1.2
**Iodine enhancement (chest)**	140	134.7	±	0.87	138.1	±	1.1	137.7	±	1.3
**Calcium (calcification)**	198	183.4	±	1.30	190.7	±	0.9	190.5	±	1.3
**Calcium (bone)**	334	315.3	±	1.20	325.6	±	0.8	324.0	±	0.6
**Iodine (15 mg/mL)**	356	322.6	±	1.58	332.5	±	0.7	330.2	±	2.1

HU, manufacturer-provided Hounsfield unit values for the object at 120 kVp.

Table S4: CT number standard deviation (noise) measurements for objects within the multi-energy CT phantom for the existing CT scanner (Standard reconstruction) and updated CT scanner (Standard and Soft reconstructions).

Object	HU	Existing-Standard			Updated-Standard			Updated-Soft		
**Adipose**	-100	12.2	±	0.20	12.5	±	0.4	10.8	±	0.5
**Brain**	15	13.9	±	0.93	13.7	±	1.2	11.5	±	0.5
**Soft tissue**	35	14.5	±	0.44	12.9	±	0.6	10.1	±	0.5
**Soft tissue**	35	15.8	±	0.80	14.1	±	0.8	12.0	±	0.8
**Soft tissue**	35	17.1	±	0.49	15.7	±	0.5	13.1	±	0.8
**Blood**	40	13.8	±	0.70	14.7	±	0.5	11.9	±	0.6
**Iodine (2 mg/mL)**	51	14.5	±	0.75	14.6	±	0.4	11.9	±	0.3
**Blood clot (normal)**	70	14.0	±	0.36	13.9	±	0.5	11.2	±	0.2
**Iodine enhancement (neuro)**	90	11.7	±	1.79	12.1	±	0.1	9.7	±	0.6
**Blood clot (extreme)**	100	12.5	±	0.78	12.0	±	0.7	9.9	±	0.6
**Iodine (5 mg/mL)**	128	15.7	±	0.31	14.8	±	1.0	12.0	±	0.6
**Iodine enhancement (chest)**	140	16.4	±	0.80	15.8	±	1.0	12.6	±	0.6
**Calcium (calcification)**	198	16.5	±	0.51	22.2	±	0.7	13.6	±	1.0
**Calcium (bone)**	334	13.3	±	0.57	24.0	±	1.6	10.8	±	0.5
**Iodine (15 mg/mL)**	356	15.3	±	0.93	27.0	±	2.3	11.7	±	1.3

HU, manufacturer-provided Hounsfield unit values for the object at 120 kVp.

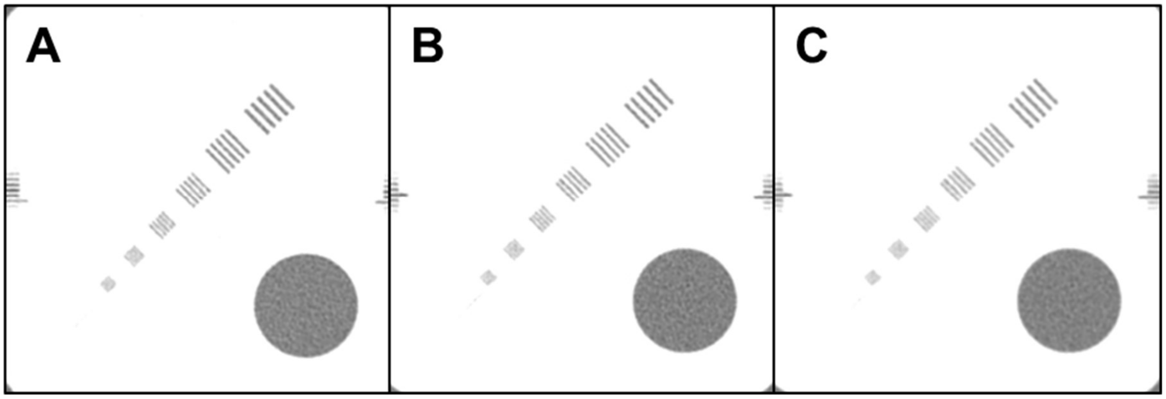

Figure S1: Results of the spatial resolution assessment using the vendor-provided quality control phantom. Results were similar between images from the A) existing and B) updated scanners reconstructed with the Standard algorithm. C) Reconstruction with the Soft algorithm on the updated scanner slightly worsened the spatial resolution though the limiting resolution was defined by the same pattern group (#4) for all images.
