# Supplementary material for: Quantification and homogenization of image noise between two CT scanner models
Source: J Appl Clin Med Phys. 2019 Dec 20;21(1):174–8. doi: 10.1002/acm2.12798 (PMC6964752; doi:10.1002/acm2.12798)
Supplement: Supplementary file 2 [file ACM2-21-174-s002.pdf]

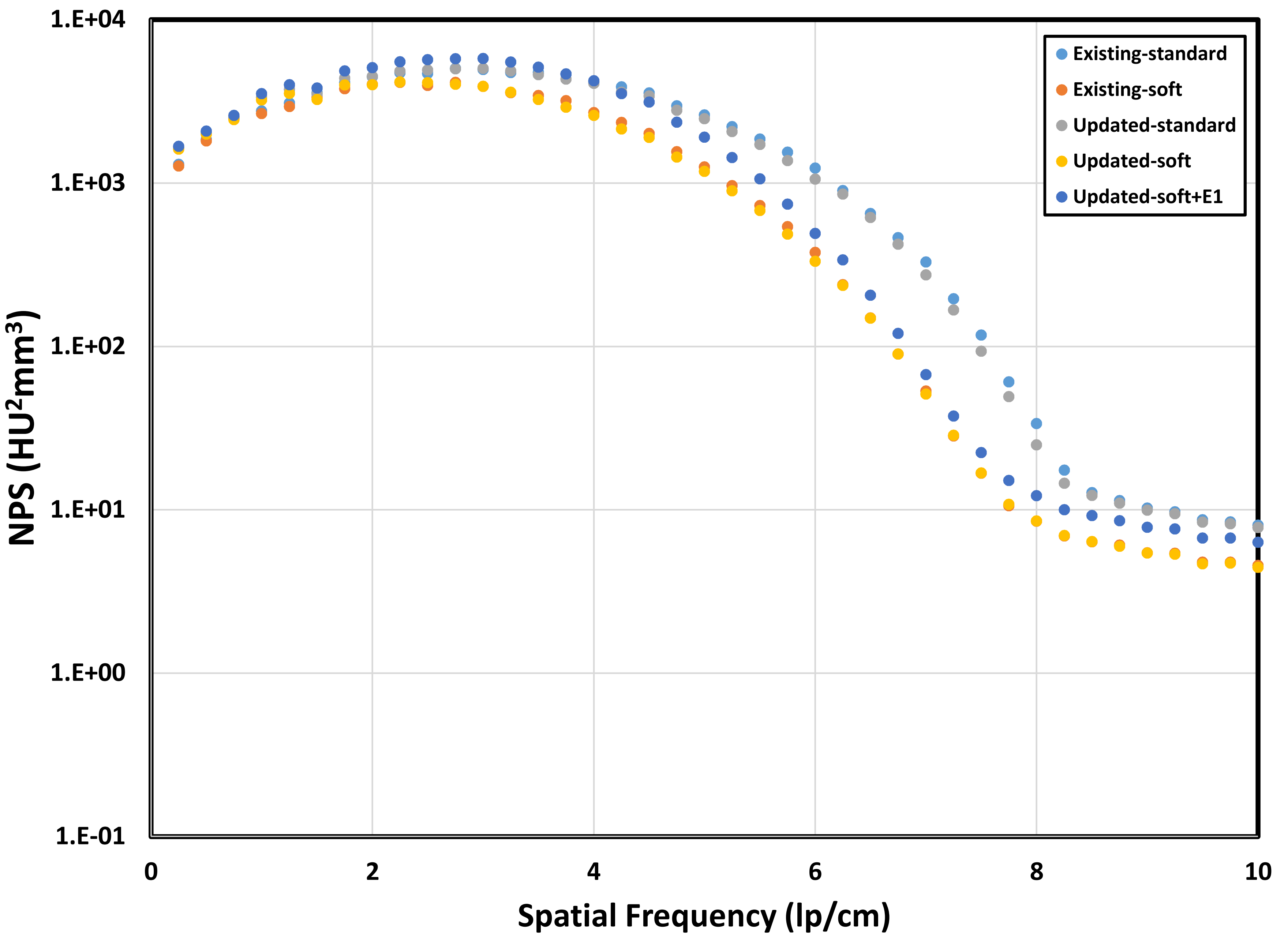

NPS (HU^2mm^3)
Spatial Frequency (lp/cm)
1.E+04
1.E+03
1.E+02
1.E+01
1.E+00
1.E-01
0
2
4
6
8
10
Existing-standard
Existing-soft
Updated-standard
Updated-soft
Updated-soft+E1

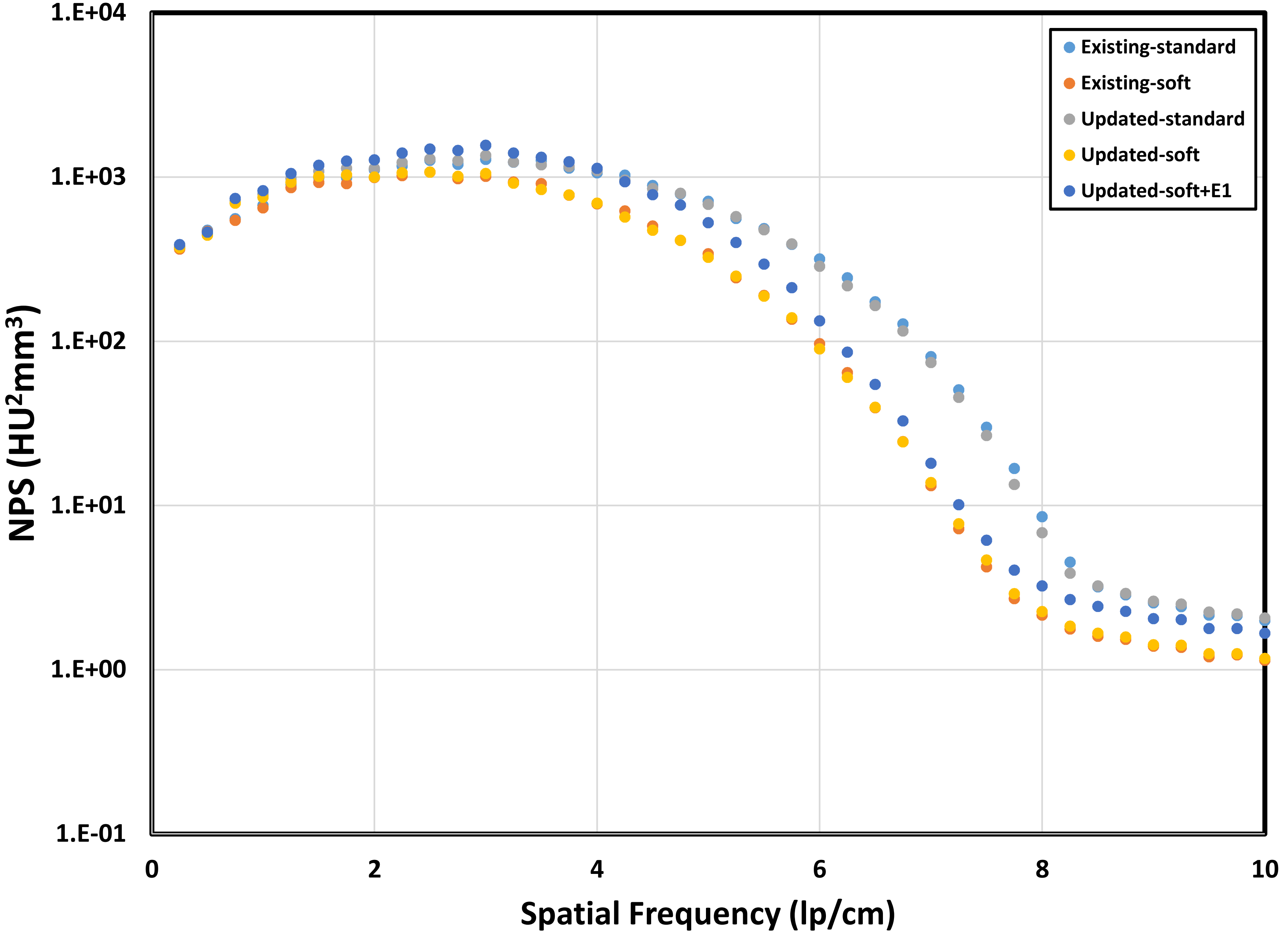

NPS (HU²mm³)
1.E+04
1.E+03
1.E+02
1.E+01
1.E+00
1.E-01
0
2
4
6
8
10
Spatial Frequency (lp/cm)
Existing-standard
Existing-soft
Updated-standard
Updated-soft
Updated-soft+E1

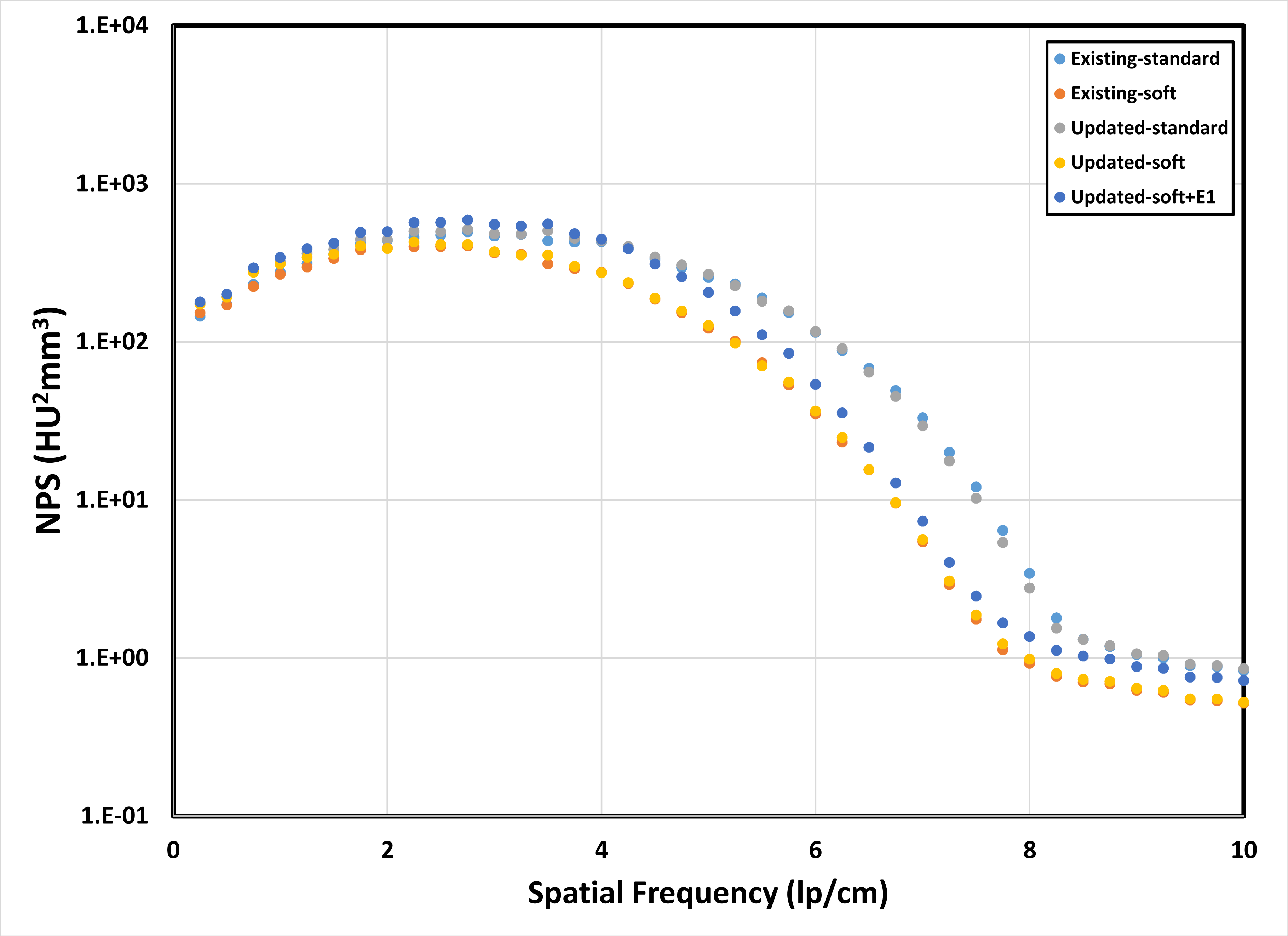

1.E+04
1.E+03
1.E+02
1.E+01
1.E+00
1.E-01
NPS (HU²mm³)
0
2
4
6
8
10
Spatial Frequency (lp/cm)
Existing-standard
Existing-soft
Updated-standard
Updated-soft
Updated-soft+E1

4.99 mGy

Scanner	Existing	Existing	Updated	Updated	Updated
Recon	standard	soft	standard	soft	soft+E1
MAX NPS	5040	4127	5037	4160	5790
Freq @ Max	3	2.25	3	2.25	3

19.96 mGy

Scanner	Existing	Existing	Updated	Updated	Updated
Recon	standard	soft	standard	soft	soft+E1
MAX NPS	1279	1070	1352	1070	1560
Freq @ Max	3	2.5	3	2.5	3

49.89 mGy

Scanner	Existing	Existing	Updated	Updated	Updated
Recon	standard	soft	standard	soft	soft+E1
MAX NPS	496	404	515	428	592
Freq @ Max	2.75	2.75	2.75	2.25	2.75
